# Non-canonical role of wild-type SEC23B in the cellular stress response pathway

**DOI:** 10.1038/s41419-021-03589-9

**Published:** 2021-03-22

**Authors:** Lamis Yehia, Darren Liu, Shuai Fu, Pranav Iyer, Charis Eng

**Affiliations:** 1 Genomic Medicine Institute, Lerner Research Institute, Cleveland Clinic, Cleveland, OH USA; 2 Cleveland Clinic Lerner College of Medicine, Cleveland, OH USA; 3 Department of Genetics and Genome Sciences, Case Western Reserve University School of Medicine, Cleveland, OH USA; 4 Taussig Cancer Institute, Cleveland Clinic, Cleveland, OH USA; 5 Germline High Risk Cancer Focus Group, Case Comprehensive Cancer Center, Case Western Reserve University, Cleveland, OH USA; 6 Present Address: Department of Mechanical and Aerospace Engineering, Princeton University, Princeton, NJ USA

**Keywords:** Cell biology, Cancer genetics

## Abstract

While germline recessive loss-of-function mutations in *SEC23B* in humans cause a rare form of anaemia, heterozygous change-of-function mutations result in increased predisposition to cancer. *SEC23B* encodes SEC23 homologue B, a component of coat protein complex II (COPII), which canonically transports proteins from the endoplasmic reticulum (ER) to the Golgi. Despite the association of *SEC23B* with anaemia and cancer, the precise pathophysiology of these phenotypic outcomes remains unknown. Recently, we reported that mutant SEC23B has non-canonical COPII-independent function, particularly within the ER stress and ribosome biogenesis pathways, and that may contribute to the pathobiology of cancer predisposition. In this study, we hypothesized that wild-type SEC23B has a baseline function within such cellular stress response pathways, with the mutant protein reflecting exaggerated effects. Here, we show that the wild-type SEC23B protein localizes to the nucleus in addition to classical distribution at the ER/Golgi interface and identify multiple putative nuclear localization and export signals regulating nuclear–cytoplasmic transport. Unexpectedly, we show that, independently of COPII, wild-type SEC23B can also localize to cell nucleoli under proteasome inhibition conditions, with distinct distribution patterns compared to mutant cells. Unbiased proteomic analyses through mass spectrometry further revealed that wild-type SEC23B interacts with a subset of nuclear proteins, in addition to central proteins in the ER stress, protein ubiquitination, and EIF2 signalling pathways. We validate the genotype-specific differential SEC23B–UBA52 (ribosomal protein RPL40) interaction. Finally, utilizing patient-derived lymphoblastoid cell lines harbouring either wild-type or mutant *SEC23B*, we show that SEC23B levels increase in response to ER stress, further corroborating its role as a cellular stress response sensor and/or effector. Overall, these observations suggest that SEC23B, irrespective of mutation status, has unexplored roles in the cellular stress response pathway, with implications relevant to cancer and beyond that, CDAII and normal cell biology.

## Introduction

Disparate disorders can be associated with dysregulated function of a single gene, with well-documented examples being the *RET* proto-oncogene in multiple endocrine neoplasia type 2 and Hirschsprung disorder and the *PTEN* tumour-suppressor gene in Cowden syndrome (CS) and autism spectrum disorder^1–4^. Such disorders provide an excellent model for uncovering previously unknown functions for known genes. Here, we focus on *SEC23B* (MIM 610512), encoding SEC23 homologue B, a component of coat protein complex II (COPII), which functions in the anterograde transport of proteins from the endoplasmic reticulum (ER) to the Golgi apparatus^5,6^. Germline loss-of-function homozygous or compound heterozygous *SEC23B* mutations cause a rare disorder, congenital dyserythropoietic anaemia type II (CDAII [MIM 224100])^7,8^, which is associated with decreased SEC23B protein levels. In humans, CDAII is characterized by anaemia and increased bi-/multi-nucleated erythroblasts in the bone marrow^7^. In contrast, we recently identified *SEC23B* as a candidate cancer-predisposition gene associated with CS (MIM 158350) and apparently sporadic thyroid cancer^9^. CS is an autosomal-dominant hereditary disorder characterized by an increased predisposition to breast, thyroid, and other cancers^10,11^ and hence serves as a useful model for cancer initiation. In this context, we identified that the germline heterozygous variants did not affect SEC23B protein levels^9^ as is observed in CDA II^12^, suggesting “change-of-function” effects.

ER stress response plays a fundamental role in regulating the balance between cell death and survival^13^. Disturbances in ER homoeostasis, such as the accumulation of misfolded proteins, result in the activation of the unfolded protein response (UPR), an evolutionarily conserved adaptive signalling cascade that aims at restoring ER function^13^. The overall downstream response of activation of the UPR is to attenuate global protein synthesis, selectively enhance the synthesis of chaperone proteins to aid in correcting misfolded proteins, activate ER-associated protein degradation to alleviate ER load, and other pro-survival mechanisms^13,14^. If the ER stress is not resolved, damage accumulates and cells activate apoptotic signalling pathways. Intriguingly, mouse models completely deficient of SEC23B do not show an anaemia phenotype but die shortly after birth and show ER stress-induced degeneration of secretory tissues, such as the pancreas and salivary glands^15^. While extensive insights have been derived from studying various model organisms, the precise mechanisms behind the cellular and molecular phenotypes in CDAII remain challenging to uncover in humans^16^.

Relatedly, it has been well documented in different human cancers that ER stress and the associated UPR signalling regulate different stages of carcinogenesis, from initiation to progression to metastasis^17,18^. Indeed, we identified that CS-related mutant SEC23B localized to cell nucleoli and associated with ER stress “addiction” and a non-canonical role within the ribosome biogenesis pathway^19^. However, what remains elusive is whether wild-type SEC23B protein has such a non-canonical role within the cellular stress response pathway irrespective of mutation status.

## Materials and methods

### Cell lines and culture conditions

The Nthy-ori 3-1 human thyroid follicular epithelium cell line (catalogue number EC90011609, lot number 09C008, passage number 16, purchased in 2014 from Sigma-Aldrich, St. Louis, MO, USA) was cultured in RPMI-1640 supplemented with 2 mM glutamine and 10% foetal bovine serum (FBS). HEK293 cells (originally purchased from the American Type Culture Collection in 2011 and obtained in 2014 from the Cleveland Clinic Lerner Research Institute Cell Culture Core) were cultured in Dulbecco’s modified Eagle’s medium supplemented with 10% FBS and 1% penicillin and streptomycin. Patient-derived immortalized lymphoblastoid cell lines (LBLs) were generated by the Genomic Medicine Biorepository of the Genomic Medicine Institute of the Cleveland Clinic (Cleveland, OH, USA) according to standard procedures and subsequently maintained in RPMI-1640 supplemented with 20% FBS and 1% penicillin/streptomycin. LBL pools were generated by co-culturing an equal number of cells from each patient according to genotype (wild-type or mutant *SEC23B*). Usage of patient-derived LBLs was approved by the Cleveland Clinic Institutional Review Board (IRB protocol *PTEN*-8458). All cell lines were maintained at 37 °C and 5% CO_2_ culture conditions and tested negative upon routine mycoplasma testing at the Eng laboratory (luminescence ratios <0.9) using the MycoAlert Mycoplasma Detection Kit (Lonza, Allendale, NJ, USA). The Nthy-ori 3-1 cell line was authenticated through STR PCR (AmpFLSTR SGM Plus PCR Amplification Kit, Life Technologies) by the European Collection of Cell Cultures (ECACC, original source of the cell line, test date 14/04/2009). Stable Nthy-ori 3-1 cell lines (Nthy-EGFP, Nthy-*SEC23B*-WT, Nthy-*SEC23B*-V594G) were generated as we had previously reported^9^. We used 4 different pools of cells at passage number 28 after selection. All experiments were conducted using cells at passage numbers between 29 and 42^9^.

### Nuclear–cytoplasmic fractionation

We followed a standard optimized protocol for nuclear–cytoplasmic fractionation from freshly harvested cells^20^. We prepared the hypotonic lysis buffer (HLB) with final component concentrations of 10 mM Tris (pH 7.5), 10 mM NaCl, 3 mM MgCl_2_, 0.3% (vol/vol) NP-40, and 10% (vol/vol) glycerol. We prepared the nuclear lysis buffer (NLB) with final component concentrations of 20 mM Tris (pH 7.5), 150 mM KCl, 3 mM MgCl_2_, 0.3% (vol/vol) NP-40, and 10% (vol/vol) glycerol. Both buffer solutions were prepared using nuclease-free water. All steps of this protocol are performed on ice and using a centrifuge cooled down to 4 °C. We resuspended the cell pellet by gentle pipetting with 1 ml of ice-cold HLB (this volume is optimized for 10 million harvested cells). The cell suspension was supplemented with a cocktail of protease and phosphatase inhibitors (Sigma-Aldrich, St. Louis, MO, USA). We incubated the cells on ice for 10 min, followed by brief vortexing. We then centrifuge the cell suspension at 800 × *g* at 4 °C for 8 min. The resulting supernatant represents the cytoplasmic fraction, which was then transferred to a new tube on ice. We added 5 M NaCl to this cytoplasmic suspension to result in a concentration of 140 mM and mixed gently through inversion. We washed the nuclear pellet that was retained after the initial centrifugation four times with HLB, pipetting gently, and centrifuging at 200 × *g* at 4 °C for 2 min. These conditions were appropriate for the Nthy-ori 3-1 and HEK293 cells. To determine appropriate washing of the nuclei, we dropped few microliters of the nuclear extract unto a glass coverslip and observed under a light microscope to evaluate any ER remnants still attached to the nuclei. The latter step is important to accordingly optimize further washing. To prepare the total nuclear extracts, we resuspended the HLB-washed nuclei in 0.5 ml of ice-cold NLB (this volume is optimized for 10 million harvested cells). The nuclear suspension was supplemented with a cocktail of protease and phosphatase inhibitors (Sigma-Aldrich). We sonicated the nuclei four times at 20% power for 15 s in an ice bath with 2 min of cooling between each sonication. We checked sonication success through observing several microliters of lysed nuclei on a glass coverslip with a light microscope. We centrifuged the cytoplasmic and nuclear fractions in 1.5 ml microcentrifuge tubes at 18,000 × *g* for 15 min at 4 °C. The supernatants were subsequently transferred to fresh tubes and quantified with the Pierce BCA Protein Assay Kit (Thermo Fisher Scientific). We loaded an equivalent mass of protein (20 μg) in each lane for the nuclear and cytoplasmic fractions. Cell lysates were separated by sodium dodecyl sulfate-polyacrylamide gel electrophoresis (SDS-PAGE) and transferred onto nitrocellulose membranes. We probed for SEC23B (Abcam ab151258) at 1:1000, SEC13 (Santa Cruz F-6, sc-514308) at 1:1000, glyceraldehyde 3-phosphate dehydrogenase (GAPDH; cytoplasmic marker, Cell Signaling #2118) at 1:20,000, PARP1 (nuclear marker, Santa Cruz F-2, sc-8007) at 1:1000, Lamin A/C (nuclear marker, Cell Signaling #2032) at 1:1000, and CANX (ER marker, Cell Signaling #2679) at 1:1000 dilutions. Blots were scanned digitally and quantified using the Odyssey Infrared Imaging System (Li-Cor Biosciences, Lincoln, NE, USA).

### Immunoblotting

Protein was extracted from whole-cell lysates with the Mammalian Protein Extraction Reagent M-PER (Thermo Fisher Scientific, Waltham, MA, USA) supplemented with a cocktail of protease and phosphatase inhibitors (Sigma-Aldrich) and was quantified with the Pierce BCA Protein Assay Kit (Thermo Fisher Scientific). Cell lysates were separated by SDS-PAGE and transferred onto nitrocellulose membranes. We probed for SEC23B (Abcam ab151258) at 1:1000, UBA52 (Abcam ab109227) at 1:1000, GAPDH (Cell Signaling #2118) at 1:20,000, and α-actinin (Cell Signaling #3134) at 1:1000 dilutions. Blots were scanned digitally and quantified using the Odyssey Infrared Imaging System (Li-Cor Biosciences).

### Immunoprecipitation

Cells were pelleted and lysed with M-PER (Thermo Fisher Scientific) supplemented with a cocktail of protease and phosphatase inhibitors (Sigma-Aldrich). Protein lysates were collected by centrifugation at 13,000 RPM for 10 min at 4 °C and pre-cleared by incubation with Protein A/G Dynabeads (Thermo Fisher Scientific) for 3 h at 4 °C on a rotator. Pre-cleared protein lysates were quantified with the Pierce BCA Protein Assay Kit (Thermo Fisher Scientific), and 1 mg/ml lysates were prepared. We used anti-GFP (Abcam ab290) for pulldown at the recommended dilution. Cell lysates were separated by SDS-PAGE, and following transfer onto nitrocellulose membranes, we probed for GFP (Abcam ab290) at 1:2000, SEC23B (Abcam ab151258) at 1:1000, and UBA52 (Abcam ab109227) at 1:1000 dilutions.

### Bioinformatic identification of putative SEC23B nuclear localization and export signals

We utilized two independent tools to predict the presence of one or more nuclear localization signals (NLSs) in the SEC23B protein. Those included cNLS Mapper^21^, which predicts importin α-dependent NLSs, and NucPred^22^, which uses an evolutionary machine learning approach to predict whether the input protein spends any time in the nuclear compartment or is entirely cytoplasmic. To predict for the existence of nuclear export signal (NES), we utilized NetNES^23^, which specifically evaluates the presence of leucine-rich NES in eukaryotic proteins. For all analyses, we used the human SEC23B primary protein sequence from Uniprot (Q15437, SC23B_HUMAN).

### Experimental identification of putative SEC23B NLS and NES

EGFP-tagged wild-type SEC23B plasmid (pMSCV backbone vector) was used to generate three truncation mutants: EGFP-SEC23B-ΔNLS1, EGFP-SEC23B-ΔNLS2, and EGFP-SEC23B-ΔNES. Wild-type plasmids were mutagenized for the truncations of interest with the QuikChange II Site-Directed Mutagenesis Kit (Agilent Technologies, Santa Clara, CA, USA) or using the restriction enzymes SalI and NotI (New England Biolabs Inc., Ipswich, MA, USA). Primer sequences used to generate the constructs are listed in Table S1. All truncation expression constructs were validated by Sanger sequencing prior to transfection. For transient overexpression, cell lines were transfected with Lipofectamine 3000 (Invitrogen, Carlsbad, CA, USA) according to the manufacturer’s instructions. Cells were grown for at least 24 h following transfection before downstream microscopic visualization of protein localization. Slides were visualized and images were obtained using a laser scanning confocal TCS SP8 microscope controlled by the Leica Application Suite Software (Buffalo Grove, IL, USA). All images were obtained using a ×63 oil immersion objective and a 488-nm laser.

### Cell treatments

Stable cell lines Nthy-EGFP, Nthy-*SEC23B*-WT, and Nthy-*SEC23B*-V594G were treated with the proteasome inhibitors MG132 (10 μM, Sigma-Aldrich) and Bortezomib (250 nM, Cell Signaling #2204) for 24 h. LBLs were treated with ER stress-inducing agent Thapsigargin (600 nM, Sigma-Aldrich) for 5 days. All drugs were reconstituted in dimethyl sulfoxide (Sigma-Aldrich).

### Immunofluorescence

We performed immunofluorescence as we have previously reported^19^. Primary antibodies include anti-SEC24C (Cell Signaling #14676) at 1:100, anti-UBF (Santa Cruz sc-13125) at 1:200, anti-Nucleophosmin/B23 (Abcam ab10530) at 1:500, and anti-Fibrillarin (Cell Signaling # 2639) at 1:400 dilutions. We used the Alexa 555 goat anti-mouse (Cell Signaling #4409) or goat anti-rabbit (Cell Signaling #4413) secondary antibodies at 1:1000 dilution for 1 h at room temperature. We performed aggresome staining using the PROTEOSTAT® Aggresome Detection Kit (Enzo Life Sciences, Farmingdale, NY, USA). Coverslips were mounted using ProLong Gold Antifade mountant with DAPI (Invitrogen). Slides were visualized and images obtained using a laser scanning confocal TCS SP8 microscope controlled by the Leica Application Suite Software (Leica). All images were obtained using a ×63 oil immersion objective and 405-, 488-, and 594-nm lasers.

### Mass spectrometry

We first performed immunoprecipitation on lysates from Nthy-EGFP, Nthy-SEC23B-WT, and Nthy-SEC23B-V594G cells, using the same protocol as stated above. We used 10 mg total protein prepared as 2 mg/ml lysates. We used 7 µg anti-GFP (Abcam ab290) and 1 µg normal rabbit IgG (Cell Signaling #3900) for pulldown. Lysates were separated by SDS-PAGE and gels were stained using SimplyBlue™ SafeStain (Thermo Fisher Scientific) following the standard manufacturer protocol. Mass spectrophotometry was performed at the Cleveland Clinic Lerner Research Institute Proteomics and Metabolomics Core (Cleveland, OH, USA). Data were analysed by using all CID spectra collected in the experiment to search against the human UniProtKB database with the programs SEQUEST^24^ and Mascot (Matrix Science Limited, London, UK). Peptide and protein identification were validated with the program Scaffold (Proteome Software Inc., Portland, OR, USA) to a false discovery rate of 1%. All matched MS2 spectra were filtered based on the Xcorr score versus charge state (default parameters). We performed pathway analysis using Ingenuity Pathway Analysis (IPA, QIAGEN Bioinformatics, Redwood City, CA, USA). *P* values derived from IPA indicate significance after Benjamini–Hochberg multiple testing correction. Spectral counts were square root transformed to reduce right-skewness of the data. We generated heatmaps using the pheatmap package (v1.0.12) using R (v3.6.2).

## Results

### Wild-type SEC23B localizes to the nucleus

**Fig. 1:**
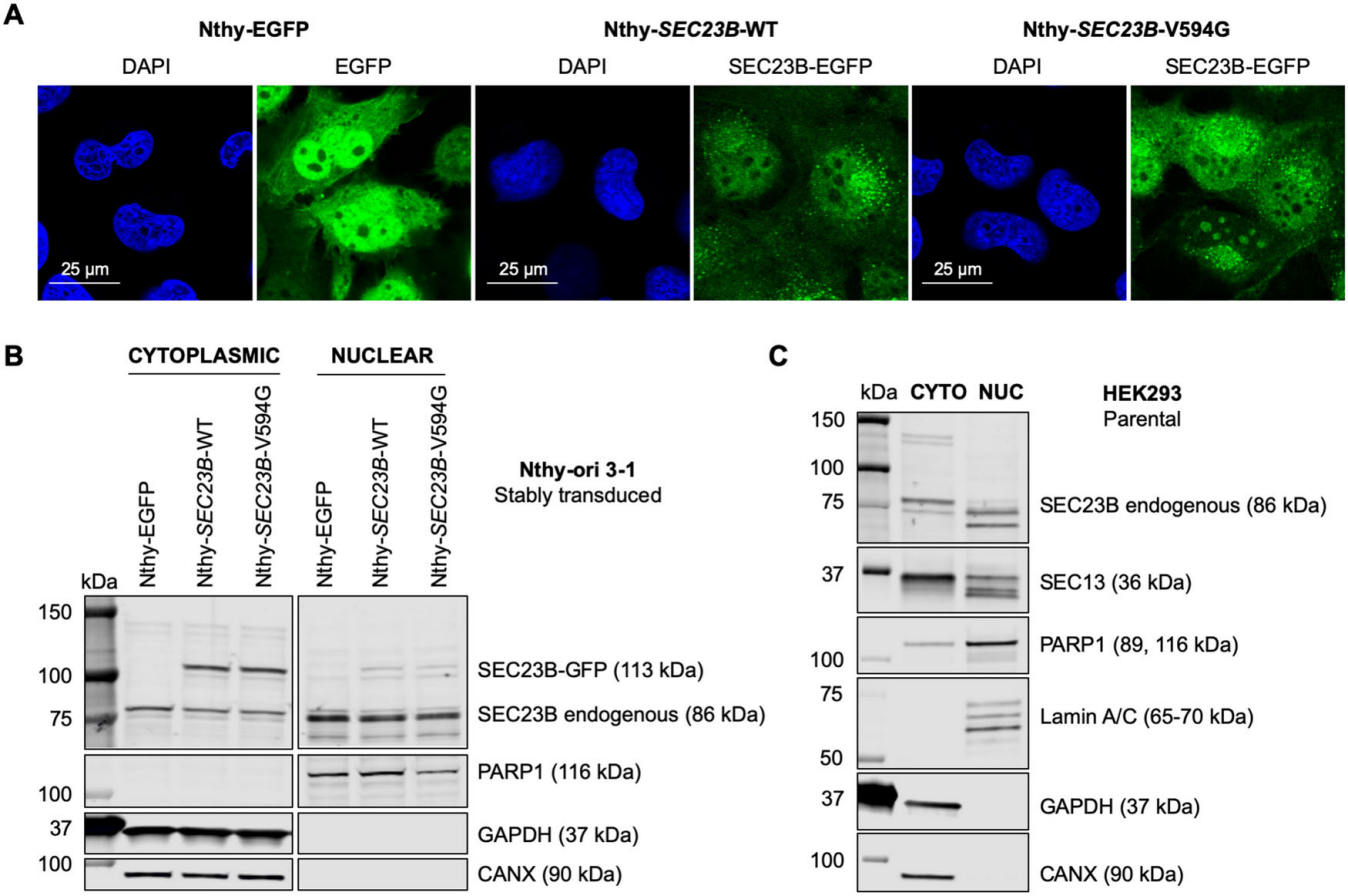
Wild-type SEC23B localizes to the nucleus. **A** Non-malignant thyroid Nthy-ori 3-1 cells were stably transduced with EGFP, wild-type *SEC23B*, and mutant p.Val594Gly *SEC23B* (the latter two fused with EGFP). Nthy-*SEC23B*-WT and Nthy-*SEC23B-*V594G cells show nuclear expression of SEC23B. Nthy-EGFP cell line is used as a control to show non-specific GFP subcellular localization. Blue, DAPI; green, SEC23B-EGFP or EGFP. Scale bars, 25 μm. **B** Protein levels after nuclear–cytoplasmic fractionation in stably transduced Nthy-ori 3-1 cells detects endogenous and EGFP-tagged SEC23B in the nuclear compartment. Nthy-EGFP cell line is used as a control to indicate baseline endogenous levels of SEC23B. PARP1, nuclear marker; GAPDH, cytoplasmic marker; CANX, endoplasmic reticulum marker. **C** Protein levels after nuclear–cytoplasmic fractionation in parental HEK293 cells detects endogenous SEC23B in the nuclear compartment. PARP1 and Lamin A/C, nuclear markers; GAPDH, cytoplasmic marker; CANX, endoplasmic reticulum marker.

SEC23B canonically localizes to the ER–Golgi as a component of the COPII-coated vesicles for ER to Golgi protein transport^5^. Previously, we reported that mutant SEC23B localizes to the nucleolus independent of COPII to impact the ribosome biogenesis pathway particularly under ER stress conditions^19^. We hypothesized that wild-type SEC23B plays a baseline role in the cellular ER stress pathway, with mutant SEC23B reflecting exaggerated effects within this pathway. To address this hypothesis, we first investigated whether wild-type SEC23B can localize to the nucleus, similar to the mutant SEC23B protein^19^. We utilized stable thyroid follicular epithelial cell lines expressing EGFP-tagged wild-type or p.Val594Gly mutant SEC23B (Nthy-*SEC23B*-WT or Nthy-*SEC23B*-V594G, respectively). This cellular model recapitulates the heterozygous state observed in the germline hereditary cancer context^9^. First, immunofluorescence microscopic analysis revealed nuclear localization of both wild-type and p.Val594Gly mutant SEC23B (Figs. 1A and S1), the latter showing previously characterized nucleolar localization^19^. Second, fractionation of the nuclear and cytoplasmic compartments from these cells indicated that both the endogenous and EGFP-tagged SEC23B are detected in the nuclear compartment, in addition to its canonical presence in the cytoplasm (Fig. 1B). As a positive control, we similarly detected SEC13 in the nucleus (Fig. S2A), knowing that this component of the outer COPII coat has been shown to shuttle to the nucleus, with a subpopulation stably interacting with the nuclear pore complex (NPC)^25^. We then performed nuclear–cytoplasmic fractionation of Nthy-*SEC23B*-WT cell lysates and were able to detect the EGFP-tagged wild-type SEC23B in the nucleus, following pulldown with anti-GFP immunoglobulins and probing the membranes with anti-SEC23B (Fig. S2B). Finally, localization of wild-type endogenous SEC23B in the nucleus was recapitulated in parental HEK293 cells (Fig. 1C). These data suggest that wild-type SEC23B can localize to the nucleus in addition to its classical localization at the ER–Golgi.

### Identification of putative SEC23B nuclear localization and export signals

**Fig. 2:**
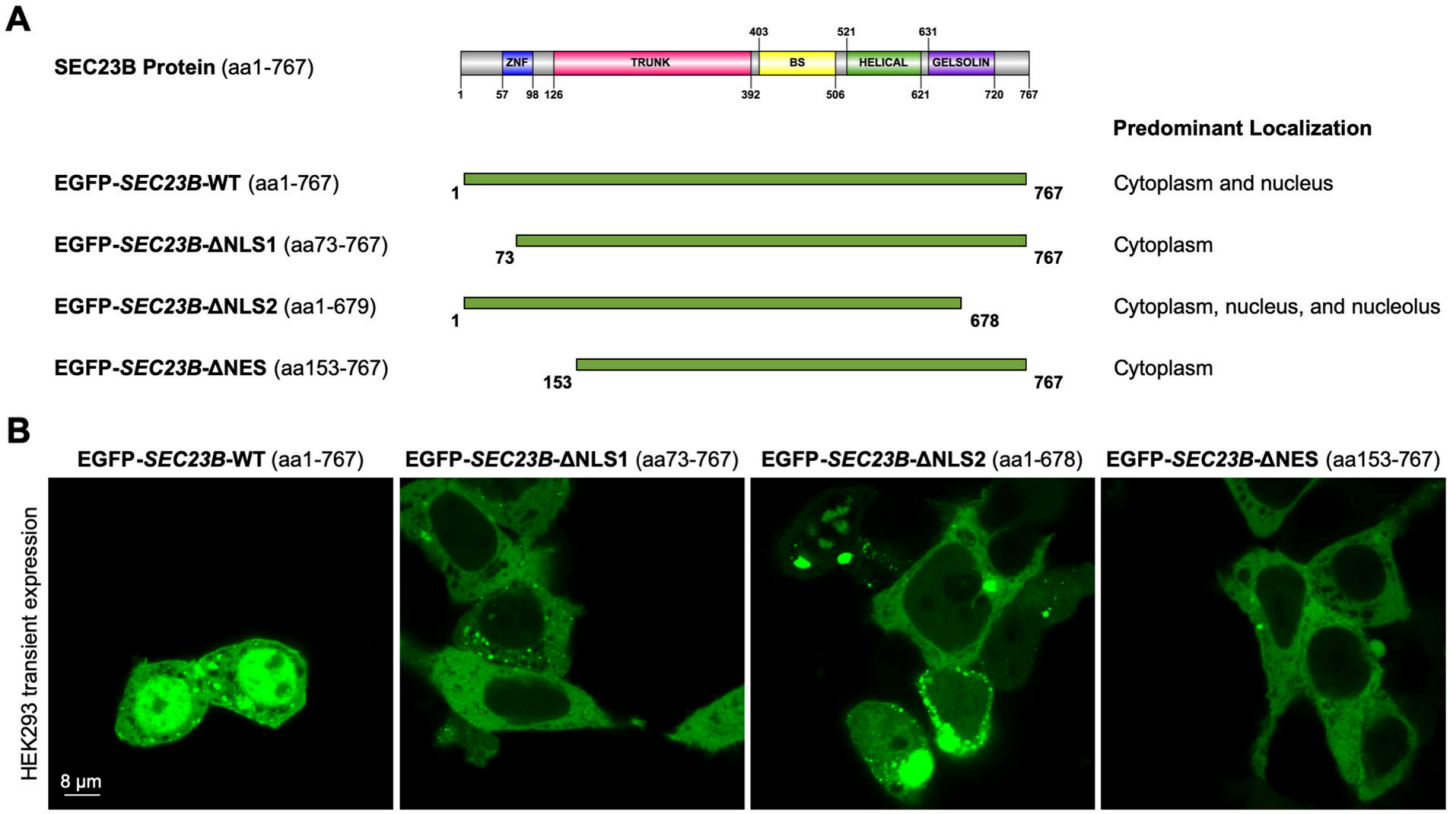
Characterization of putative SEC23B nuclear localization and export signals. **A** Truncation mutant constructs generated to characterize the bioinformatically top scoring NLS sequences and the single predicted NES sequence. The full-length SEC23B protein (Uniprot Q15437, SC23B_HUMAN) is depicted in the top panel. aa amino acids. **B** Confocal microscopic images of HEK293 cells transiently transfected with each of the constructs. The corresponding localization of the SEC23B protein is summarized in **A**. The predicted NES overlaps with the predicted NLS1 sequence. Scale bars, 8 μm.

One of the mechanisms by which proteins shuttle between the cytoplasm and the nucleus is through the classical nuclear import pathway. Through this pathway, proteins harbouring NLSs are imported into the nucleus through the NPC^26^. In order to investigate whether SEC23B protein sequence contains any NLS, we first performed bioinformatic analyses using two independent algorithms that indeed predicted dual existence of the protein within the cytoplasm and the nucleus, as well as the presence of multiple putative NLS sequences (Fig. S3A, B). We also evaluated the presence of a NES and identified a putative leucine-rich NES at amino acids 144–153 (Fig. S3C). Interestingly, this NES overlapped with one of the predicted NLS sequences (Fig. S3). Next, we generated EGFP-tagged SEC23B constructs (Fig. 2A), including the full-length protein (EGFP-*SEC23B*-WT), truncation of the two top scoring bioinformatically predicted NLS sequences (EGFP-*SEC23B*-ΔNLS1, truncating the first NLS at amino acids 43–72; EGFP-*SEC23B*-ΔNLS2, truncating the last NLS at amino acids 679–767), and truncation of the predicted NES (EGFP-*SEC23B*-ΔNES, truncating amino acids 144–152). Transient transfection of these constructs, respectively, in HEK293 cells revealed both cytoplasmic and nuclear localization of wild-type full-length SEC23B (Fig. 2B). Expectedly, truncating the two predicted NLS sequences resulted in a predominantly cytoplasmic SEC23B presence. Intriguingly, EGFP-*SEC23B*-ΔNLS2 also localized to the nucleus and nucleoli of some cells (Fig. 2B). Transfecting EGFP-*SEC23B*-ΔNES resulted in exclusively cytoplasmic SEC23B protein, presumably due to the abrogation of the overlapping NLS sequence (NLS1, Fig. 2B). Overall, these data provide evidence for the existence of NLS and NES sequences that regulate the dynamic trafficking of SEC23B between the cytoplasmic and nuclear compartments.

### Proteasome inhibition causes COPII-independent nucleolar localization of wild-type SEC23B

With the identification and validation of putative NLS and NES sequences, we next turned our attention towards investigating the dynamics of SEC23B trafficking between the cytoplasm and the nucleus. Steady-state localization of a protein appears predominantly cytoplasmic if the equilibrium of bidirectional transport across the nuclear membrane favours nuclear export. However, since wild-type SEC23B has not been previously reported to traffic to the nucleus under normal baseline conditions, we hypothesized that such a translocation event could either be due to unique biochemical changes inherent to the p.V594G-mutant protein or masked in the wild-type protein if the equilibrium of bidirectional nuclear–cytoplasmic transport favours nuclear export. To test our hypothesis that SEC23B, including wild-type protein, could translocate to the nucleus, we first treated Nthy-EGFP, Nthy-*SEC23B*-WT, and Nthy-*SEC23B*-V594G cells with Leptomycin B (LMB), a potent inhibitor of CRM1 (chromosomal maintenance 1, also known as Exportin 1) protein-mediated nuclear export^27,28^. If our hypothesis is correct, then we should observe a time-dependent accumulation of SEC23B in the nucleus. However, we only observed a subset of cells showing prominent presence of SEC23B within the nucleus (Fig. S4A).

**Fig. 3:**
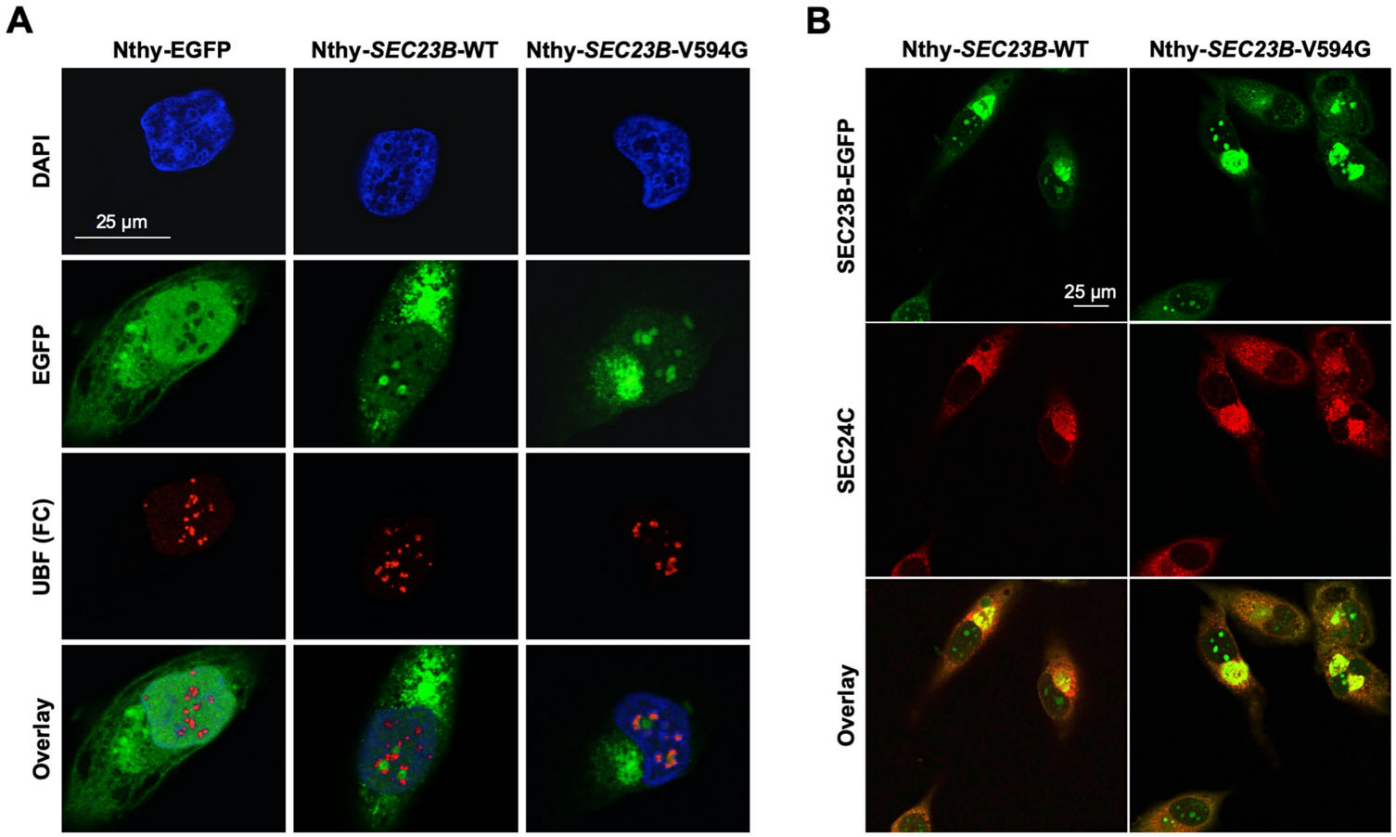
Proteasome inhibition causes COPII-independent nucleolar localization of wild-type SEC23B. **A** Immunofluorescence staining of stably transduced Nthy-ori 3-1 thyroid cells with a nucleolar protein marker. FC fibrillar centre of the nucleolus. Scale bars, 25 µm. **B** Immunofluorescence staining of Nthy-*SEC23B*-WT and Nthy-*SEC23B*-V594G cells with anti-SEC24C antibody and DAPI, followed by imaging with confocal microscopy. Scale bars, 25 µm.

Since protein stability has been shown to affect efficient protein localization^29^, we next treated cells with the proteasome inhibitor MG132, with and without LMB. Surprisingly, the presence of MG132 resulted in nuclear aggregation of SEC23B, including the wild-type protein, even in the absence of LMB (Fig. S4B). Immunofluorescence staining of nucleolar markers representing the granular component, dense fibrillar component, and fibrillar centre confirmed localization of SEC23B proximal to all regions of cell nucleoli (Figs. 3A and S5). Of note, after MG132 treatment, nucleolar cavities (dark regions within the nucleus) appear to harbour wild-type SEC23B aggregates, the latter surrounded by numerous nucleolar marker proteins such as nucleophosmin, fibrillarin, and UBF (Fig. 3A middle panel, and Fig. S5). In contrast, in addition to a similar aggregation of p.V594G SEC23B within the nucleolar cavities, a subpopulation of mutant SEC23B overlapped with the nucleolar protein markers (Fig. 3B right panel, and Fig. S5). Importantly, we show that SEC23B nucleolar presence is independent of SEC24, the COPII inner coat binding partner of SEC23^30^ (Fig. 3B). Relatedly, the nucleolar localization of SEC23B does not represent a generic and non-specific event secondary to MG132 treatment since we observed the same effect after treating the cells with Bortezomib, another proteasome inhibitor (Fig. S6). Moreover, immunostaining for p53, a nuclear protein that can translocate to the nucleolus under stress^29,31^, did not show similar effects in our cellular model (Fig. S7). Interestingly, both wild-type and mutant SEC23B co-localized with the Cowden-relevant phosphatase and tensin homologue (PTEN) in the nucleolus (Fig. S8). PTEN has been previously reported to exist in the nucleolus and to regulate nucleolar morphology and ribosome biogenesis^32–34^.

Thus far, out data suggest that independent of mutation status, SEC23B can exist in the nucleus, with preferential localization within the nucleolus under proteasome inhibition conditions or with particular mutations^19^. This localization further supports largely unexplored non-canonical and COPII-independent roles for the SEC23B protein.

### Global overview of the SEC23B interactome

**Fig. 4:**
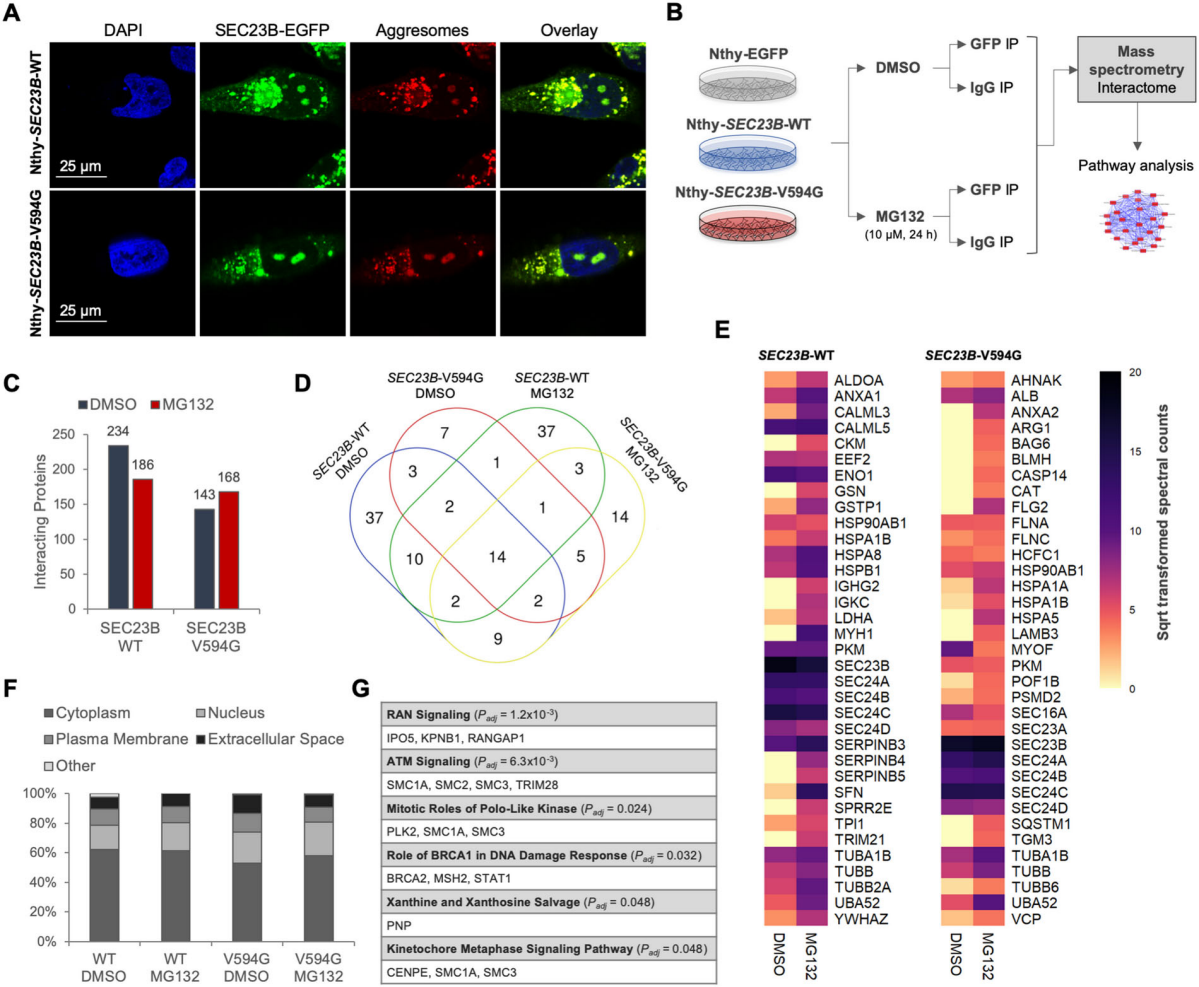
Global overview of the SEC23B interactome. **A** Immunofluorescence staining of stably transduced Nthy-ori 3-1 thyroid cells for aggresomes. Blue, DAPI; green, SEC23B-EGFP or EGFP. Scale bars, 25 µm. **B** Experimental design of the mass spectrometry experiment with and without MG132 treatment. IgG immunoglobulin, IP immunoprecipitation. **C** Global number of interacting partners in Nthy-*SEC23B*-WT and Nthy-*SEC23B*-V594G cells with and without MG132 treatment. Only protein interactants with spectral count ratios ≥2 relative to the IgG and Nthy-EGFP immunoprecipitations (negative controls) are depicted. **D** Overlapping and unique interacting partners between untreated and MG132-treated Nthy-*SEC23B*-WT and Nthy-*SEC23B*-V594G cells. Only proteins with spectral counts ≥10 are included in this Venn diagram. Table S2 includes the complete list of interacting proteins. **E** Heatmaps of the top 35 interacting proteins based on spectral counts in wild-type Nthy-*SEC23B*-WT and mutant Nthy-*SEC23B*-V594G cells. COPII component and related proteins (SEC23B, SEC23A, SEC24A-D, SEC31A, SEC23IP, SEC16A) serve as positive controls. Table S2 includes the complete list of interacting proteins. Sqrt square root. **F** Subcellular localization analysis using Ingenuity Pathway Analysis (IPA) indicates that a subset (16–23%) of the SEC23B-interacting proteins is predicted to reside in the nucleus. **G** Pathway analysis of SEC23B-interacting proteins predicted to reside in the nucleus.

The nucleolus is a cellular stress sensor and a central hub for orchestrating the stress response^35,36^. The cellular stress response pathway, such as through proteasome inhibition (i.e. lack of protein degradation and subsequent overload), typically results in the formation of “aggresomes” that include cancer-related proteins, including oncogenes and tumour-suppressor genes^37–40^. Immunofluorescence staining of misfolded and aggregated proteins (see “Materials and methods”) indeed validated that SEC23B localizes to nucleolar aggresomes in response to proteasome inhibition (Figs. 4A and S9). The fact that SEC23B can coexist with classic cancer-related proteins within nucleolar aggresomes raised the question as to whether SEC23B protein directly interacts with such pertinent proteins. Additionally, since subcellular localization and nuclear–cytoplasmic shuttling are governed by protein–protein interactions, we performed an unbiased proteomics experiment to identify the SEC23B protein interactome from non-malignant thyroid cells at baseline and after treatment with MG132 (Fig. 4B).

Through this experiment, our goals were to (1) identify whether SEC23B interacts with nuclear/nucleolar proteins, and (2) determine whether interacting partners played a known role in the cellular stress response pathway. Immunoprecipitation followed by mass spectrometry identified multiple protein interactants from Nthy-*SEC23B*-WT and Nthy-*SEC23B*-V594G cells, with and without proteasome inhibition (Fig. 4C–E and Table S2). As expected, known SEC23B-interacting proteins were identified, including the COPII component proteins SEC24A-D and SEC31A (Fig. 4E). Whereas the majority of interacting proteins are predicted to localize to the cytoplasm, a subset (16–23%) of proteins are predicted to reside in the nucleus (Fig. 4F). Examples of such nuclear proteins include structural chromosomal proteins (SMC1A, SMC2, SMC3, SMC4, MCM7), B double prime 1 (BDP1, a subunit of RNA polymerase III transcription initiation factor IIIB), RNA helicase and ATPase (UPF1), ribosomal protein L12 (RPL12), and others (Table S2). Pathway enrichment analysis identified pertinent signalling pathways such as RAN (RAS-related nuclear protein) signalling (*P*_adj_ = 1.2 × 10^−3^), which regulates nucleocytoplasmic RNA and protein transport through the NPC (Fig. 4G)^41^.

### SEC23B interacts with proteins related to the cellular stress response pathway

**Fig. 5:**
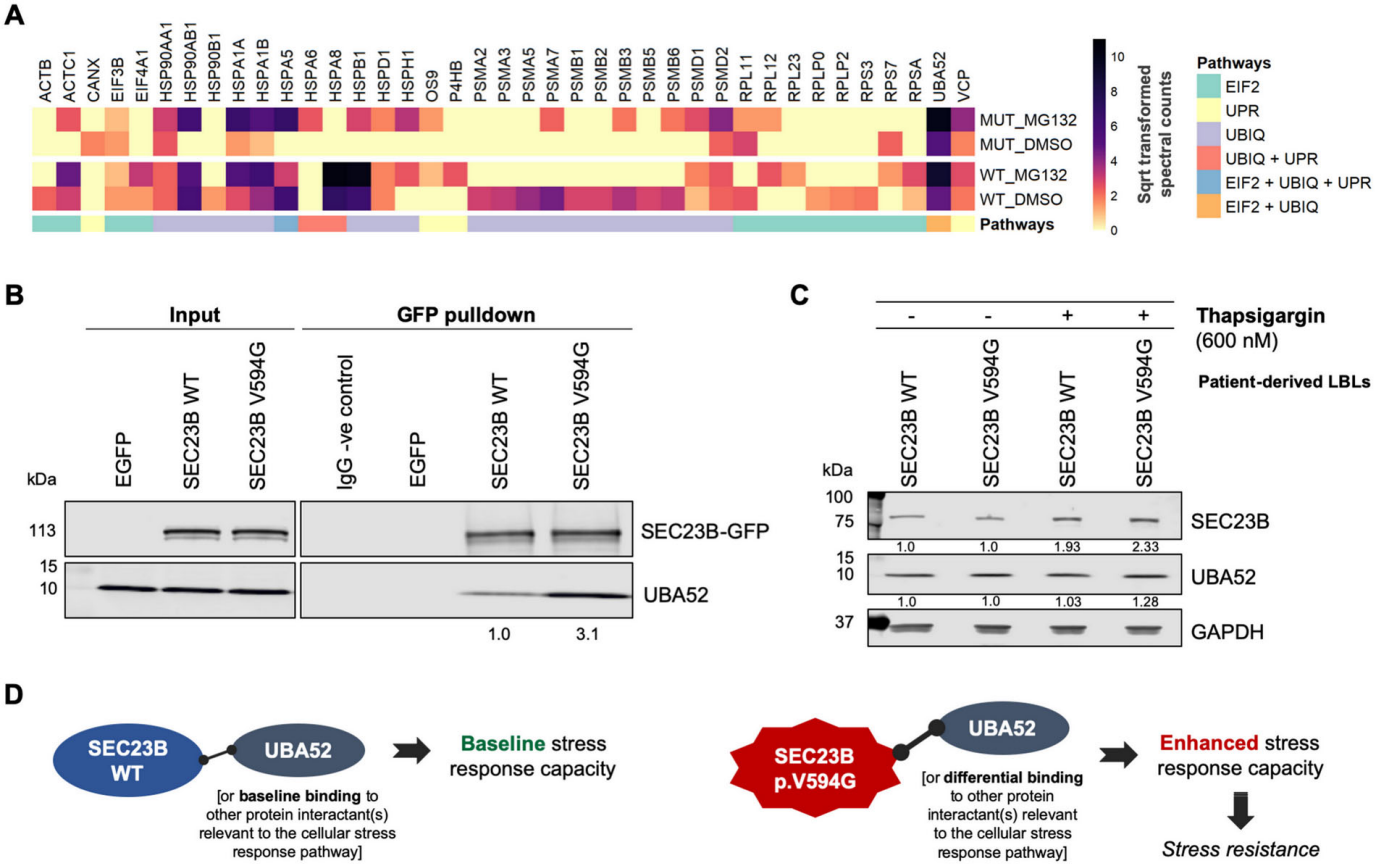
SEC23B interacts with proteins related to the cellular stress response pathway. **A** Pathway analysis of SEC23B-interacting proteins in Nthy-*SEC23B*-WT and Nthy-*SEC23B*-V594G cells with and without MG132 treatment. EIF2 eukaryotic initiation factor 2 signalling, UPR unfolded protein response, UBIQ protein ubiquitination pathway, Sqrt square root, MUT *SEC23B*-V594G. **B** Validation of the SEC23B–UBA52 interaction using immunoprecipitation of EGFP-tagged SEC23B in Nthy-*SEC23B*-WT and Nthy-*SEC23B*-V594G cells followed by immunoblotting using the UBA52 antibody. Nthy-EGFP cell line and normal rabbit IgG are used as negative controls. UBA52 protein levels are normalized to SEC23B-EGFP pulldown within each genotype and quantification values are relative to wild-type. Mutant SEC23B shows increased binding to UBA52. **C** SEC23B and UBA52 protein levels in patient-derived *SEC23B* wild-type and p.Val594Gly mutant lymphoblastoid cell line (LBL) pools with and without treatment with the ER stress-inducing agent Thapsigargin (600 nM, 5 days). Each genotypic group includes 5 LBLs with either wild-type or p.V594G mutant SEC23B, respectively (see “Materials and methods”). **D** Proposed model of how SEC23B, irrespective of mutation status, could be playing multiple unexplored roles in the cellular stress response pathway. SEC23B–UBA52 cross-talk represents one proof-of-principle example.

To gain biological insights regarding the identified SEC23B-interacting proteins, we performed pathway enrichment analysis. Notably, among the enriched canonical pathways, we identified multiple interacting proteins belonging to the protein ubiquitination pathway (*P*_adj_ = 2.2 × 10^−8^), unfolded protein response (*P*_adj_ = 5.2 × 10^−7^), and EIF2 signalling (*P*_adj_ = 2.5 × 10^−4^) (Fig. 5A). While this observation corroborates our previous findings implicating mutant SEC23B in the ER stress response and ribosome biogenesis pathway^9,19^, these pathways were also pertinent for the wild-type SEC23B protein.

Relevant to the stress response pathway and ribosome biogenesis, UBA52 was particularly germane because it is a ribosomal protein (RPL40) that has been shown to regulate selective translation of stress-related transcripts, protein synthesis, and the cell cycle^42–44^. Importantly, UBA52 also emerged as one of the top interacting partners after the COPII component and related proteins (SEC23B, SEC23A, SEC24A-D, SEC31A, SEC23IP, SEC16A), which served as a positive control. Indeed, we validate through immunoprecipitation the binding interaction between SEC23B and UBA52, with notably increased binding interaction with mutant SEC23B p.V594G (Fig. 5B).

Finally, to validate whether our observations are pertinent in CS patients, we generated pools of patient-derived LBLs (see “Materials and methods”) that are wild-type for *SEC23B* (LBL-*SEC23B*-WT) or harbouring *SEC23B* p.V594G heterozygous mutation (LBL-*SEC23B*-V594G), respectively. We then treated these cells with Thapsigargin, an ER stress-inducing agent, and observed increased SEC23B protein levels in both wild-type and mutant cells (Fig. 5C). These results validate our cellular and molecular observations that SEC23B plays a non-canonical role as a stress response effector (Fig. 5D).

## Discussion

Inherited cancer syndromes represent valuable models for the identification and characterization of both known (in an unfamiliar context) and novel cancer-predisposing genes. Since the discovery of the association of germline heterozygous *SEC23B* variants with CS and a subset of sporadic cancers^9^, multiple studies have corroborated the role of this well-known anaemia-associated gene in tumorigenesis^19,45–49^. However, the precise mechanism of these oncogenic effects, particularly in the context of the wild-type SEC23B protein, remain uncharacterized. In this study, we hypothesized that wild-type SEC23B has a baseline function within the cellular stress response pathway, with the established function of the mutant protein within this pathway representing the most exaggerated effects^19^. Hence, utilizing the mutant SEC23B protein as a point of reference, we extrapolate similarly non-canonical and unexpected functions for the wild-type SEC23B protein.

A precedent discovery of non-canonical localization and function was reported for Sec13, a component of the outer coat of COPII. A subpopulation of Sec13 shuttled to the nucleus through stable interactions with the NPC protein Nup96^25^. A genetic in vivo model developed years later demonstrated distinct roles for Sec13 at both compartments: loss of COPII function resulted in digestive organ defects, whereas loss of NPC function resulted in retinal lamination defects^50^. These data provide further support for the existing dual and tissue/context-specific roles of COPII proteins. The identification of multiple putative NLS sequences and an overlapping predicted NES sequence in the SEC23B protein suggest that the nuclear–cytoplasmic trafficking of SEC23B is likely a complex and highly regulated process that warrants detailed investigation. Relevant to CS and associated cancers, PTEN serves as a well-established model for nuclear–cytoplasmic shuttling and associated functions within each cellular compartment^3^. However, prior to such extensive characterization within the nucleus, PTEN was classically believed to be an exclusively cytoplasmic protein^51^.

We were particularly intrigued by the incidental observation that wild-type SEC23B could localize to nucleolar aggresomes after proteasome inhibition, independent of COPII. The nucleolus is a cellular stress sensor and a central hub for orchestrating the cellular stress response^35,36^. Importantly, nuclear proteins such as PML, p53, MDM2, and other proteins governing cell survival and the stress response have been shown to accumulate to nucleolar aggresomes under proteasome inhibition conditions^37–40^. Our unbiased proteomics analysis further indicated that wild-type SEC23B interacts with multiple proteins in the ER stress, protein ubiquitination, and EIF2 signalling pathways. These data corroborate our previous transcriptomic and functional analyses specific to mutant SEC23B^19^. We validate the SEC23B–UBA52 interaction with increased binding noted in mutant cells. UBA52 (ubiquitin-60S ribosomal protein L40, RPL40) is a ribosomal protein that is translationally fused to ubiquitin and that plays a role in enhancing ribosome biogenesis^52,53^. Recent studies have also shown that UBA52 regulates selective translation, including a subset of stress response transcripts^43^, and regulates protein synthesis and the cell cycle^44^. Notably, other identified SEC23B-interacting partners include 78 kDa glucose-regulated protein (GRP78, HSPA5/BiP), ER stress sensor and master regulator of the UPR^54^, and 26S proteasome non-ATPase regulatory subunit 2 (PSMD2) important for proteasome substrate binding and recognition^55^.

Importantly, we also observe an increase of SEC23B protein levels in patient-derived LBLs upon ER stress. This observation is supported by the somatic cancer context, whereby *SEC23B* copy number amplifications (including wild-type) have been identified in CS component malignancies, such as breast, endometrial, and colorectal cancers^19,56^. Whether SEC23B functions as a cellular stress response sensor or effector warrants further experimentation. Relatedly, whether alterations in SEC23B protein levels impact COPII complex-related stoichiometry towards non-canonical functions for SEC23B and the other COPII components remains to be determined. Importantly, while wild-type and mutant SEC23B share some binding partners in common, similar to UBA52, we predict that mutation status will result in differential binding interactions impacting downstream cellular phenotypes and associated signalling pathways. Notably, the UPR, protein ubiquitination, and EIF2 signalling pathways crosstalk extensively in the context of ribosome biogenesis^57–59^. In toto, therefore, our data suggest that SEC23B, irrespective of mutation status, could be playing multiple unexplored roles in the cellular stress response pathway.

## Supplementary information

Supplemental Material

Dataset 1

## Data Availability

All analyses are included in the figures, tables, and Supplementary Data files.
