# Supplementary material for: Non-canonical role of wild-type SEC23B in the cellular stress response pathway
Source: Cell Death Dis. 2021 Mar 22;12(4):304. doi: 10.1038/s41419-021-03589-9 (PMC7985502; doi:10.1038/s41419-021-03589-9)
Supplement: Supplementary file 1 — Supplemental Material [file 41419_2021_3589_MOESM1_ESM.pdf]

Figure S1: Nuclear-cytoplasmic fractionation in stably transduced Nthy-ori 3-1 cells

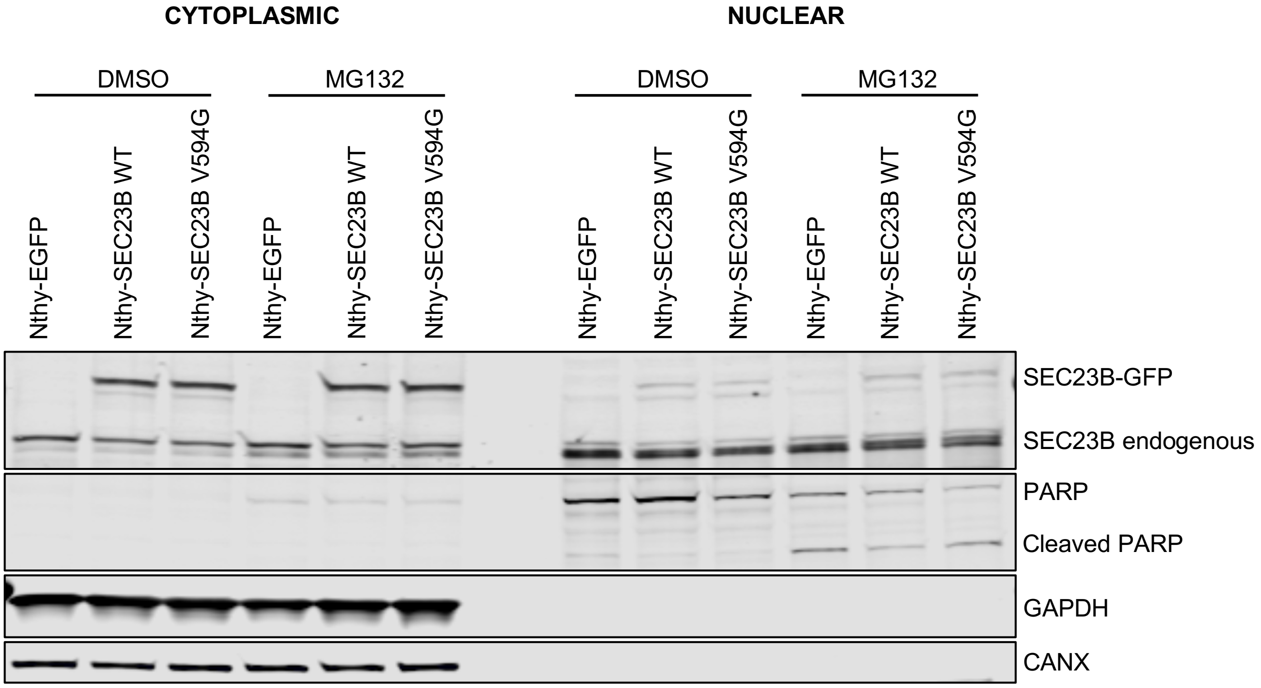

Full blot related to Figure 1B. Protein levels after nuclear-cytoplasmic fractionation in stably transduced Nthy-ori 3-1 cells detects endogenous and EGFP-tagged SEC23B in the nuclear compartment with and without MG132 treatments. Nthy-EGFP cell line is used as a control to indicate baseline endogenous levels of SEC23B. PARP1, nuclear marker; GAPDH, cytoplasmic marker; CANX, endoplasmic reticulum marker.

Figure S2: Localization of wildtype SEC23B in the nucleus

A.

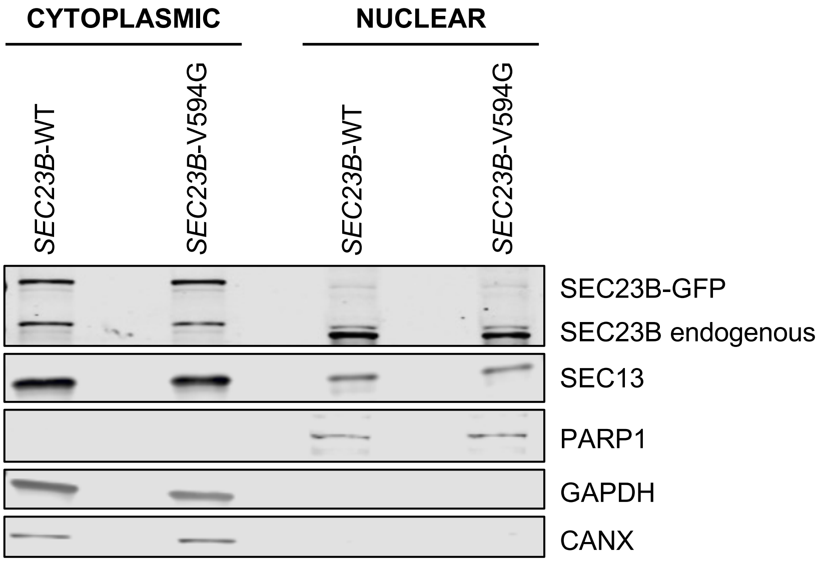

B.

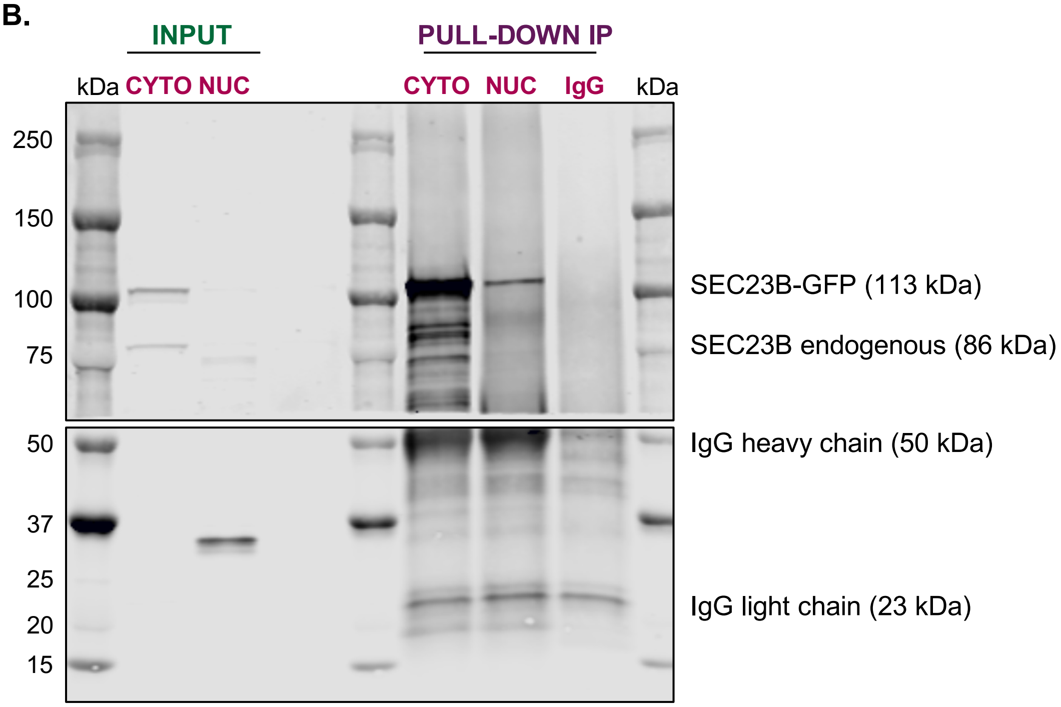

A. Nuclear-cytoplasmic (N:C) fractionation in stably transduced Nthy-ori 3-1 cells detects endogenous and EGFP-tagged SEC23B, as well as SEC13 in the nuclear compartment. PARP1, nuclear marker; GAPDH, cytoplasmic marker; CANX, endoplasmic reticulum marker.

B. N:C fractionation of Nthy-*SEC23B*-WT cell lysates followed by immunoprecipitation with anti-GFP and probing the Western blot membranes with anti-SEC23B. EGFP-tagged SEC23B is detected in the cytoplasm and nucleus. The two images represent independently scanned regions of the same nitrocellulose membrane. CYTO, cytoplasmic fraction; NUC, nuclear fraction; IgG, non-specific negative control antibody.

Figure S3: Identification of putative SEC23B nuclear localization (NLS) and export (NES) signals

A.

cNLS Mapper: Prediction of importin α-dependent nuclear localization signals (score 3-5 = localized to both nucleus and cytoplasm)

Predicted NLSs in query sequence

```
MATYLEFIQQNEERDGVRFSWNVWPSSRLEATRMVVPLACLLTPLKERPD  50
LPPVQYEPVLCSRPTCKAVLNPLCQVDYRAKLWACNFCFQRNQFPPAYGG 100
ISEVNQPAELMPQFSTIEYVIQRGAQSPLIFLYVVDTCLEEDDLQALKES 150
LQMSLSLLPPDALVGLITFGRMVQVHELSCEGISKSYVFRGTKDLTAKQI 200
QDMLGLTKPAMPMQQARPAQPQEHPFASSRFLQPVHKIDMNLTDLLGELQ 250
RDPWPVTQGKRPLRSTGVALSIAVGLLEGTFPNTGARIMLFTGGPPTQGP 300
GMVVGDELKIPIRSWHDIEKDNARFMKKATKHYEMLANRTAANGHCIDIY 350
ACALDQTGLLEMKCCANLTGGYMVMGDSFNTSLFKQTFQRIFTKDFNGDF 400
RMAFGATLDVKTSRELKIAGAIGPCVSLNVKGPCVSENELGVGGTSQWKI 450
CGLDPTSTLGIYFEVVNQHNTPIPQGGRGAIQFVTHYQHSSTQRRIRVTT 500
IARNWADVQSQLRHIEAAFDQEAAAVLMARLGVFRAESEEGPDVLRWLDR 550
QLIRLCQKFGQYNKEDPTSFRLSDSFSLYPQFMFHLRRSPFLQVFNNSPD 600
ESSYYRHHFARQDLTQSLIMIQPILYSYSFHGPPEPVLLDSSSILADRIL 650
LMDTFFQIVIYLGETIAQWRKAGYQDMPEYENFKHLLQAPLDDAQEILQA 700
RFPMPRYINTEHGGSQARFLLSKVNPSQTHNNLYAWGQETGAPILTDDVS 750
LQVFMDHLKKLAVSSAC                                  767
```

Predicted bipartite NLS

Pos.	Sequence	Score
43	TPLKERPDLPPVQYEPVLCSRPTCKAVLNP	4
43	TPLKERPDLPPVQYEPVLCSRPTCKAVLNP	3.1
143	DLQALKESLQMSLSLLPPDALVGLITFGRMV	3.2
205	GLTKPAMPMQQARPAQPQEHPFASSRFLQP	3.5
230	RFLQPVHKIDMNLTDLLGELQRDPWPVTQGKRPL	3.1
257	TQGKRPLRSTGVALSIAVGLLEGTFPNTGA	3.5
306	DELKIPIRSWHDIEKDNARFMKKATKHYEM	3.6
361	EMKCCANLTGGYMVMGDSFNTSLFKQTF	3
679	EYENFKHLLQAPLDDAQEILQARFPMPRYINTE	3.9

B.

NucPred: Score 0.4 = both cytoplasmic and nuclear

```
  1 MATYLEFIQQNEERDGVRFSWNVWPSSRLEATRMVVPLACLLTPLKERPD  50
 51 LPPVQYEPVLCSRPTCKAVLNPLCQVDYRAKLWACNFCFQRNQFPPAYGG 100
101 ISEVNQPAELMPQFSTIEYVIQRGAQSPLIFLYVVDTCLEEDDLQALKES 150
151 LQMSLSLLPPDALVGLITFGRMVQVHELSCEGISKSYVFRGTKDLTAKQI 200
201 QDMLGLTKPAMPMQQARPAQPQEHPFASSRFLQPVHKIDMNLTDLLGELQ 250
251 RDPWPVTQGKRPLRSTGVALSIAVGLLEGTFPNTGARIMLFTGGPPTQGP 300
301 GMVVGDELKIPIRSWHDIEKDNARFMKKATKHYEMLANRTAANGHCIDIY 350
351 ACALDQTGLLEMKCCANLTGGYMVMGDSFNTSLFKQTFQRIFTKDFNGDF 400
401 RMAFGATLDVKTSRELKIAGAIGPCVSLNVKGPCVSENELGVGGTSQWKI 450
451 CGLDPTSTLGIYFEVVNQHNTPIPQGGRGAIQFVTHYQHSSTQRRIRVTT 500
501 IARNWADVQSQLRHIEAAFDQEAAAVLMARLGVFRAESEEGPDVLRWLDR 550
551 QLIRLCQKFGQYNKEDPTSFRLSDSFSLYPQFMFHLRRSPFLQVFNNSPD 600
601 ESSYYRHHFARQDLTQSLIMIQPILYSYSFHGPPEPVLLDSSSILADRIL 650
651 LMDTFFQIVIYLGETIAQWRKAGYQDMPEYENFKHLLQAPLDDAQEILQA 700
701 RFPMPRYINTEHGGSQARFLLSKVNPSQTHNNLYAWGQETGAPILTDDVS 750
751 LQVFMDHLKKLAVSSAC                                  767
```

Positively and negatively influencing subsequences are coloured according to the following scale:

(non-nuclear) negative ||||||||||||||||||||||||||||||||||||||||||||||||||| positive (nuclear)

C.

NetNES: Predicts leucine-rich NES at amino acids 144-153

>Sequence - NetNES 1.1 prediction

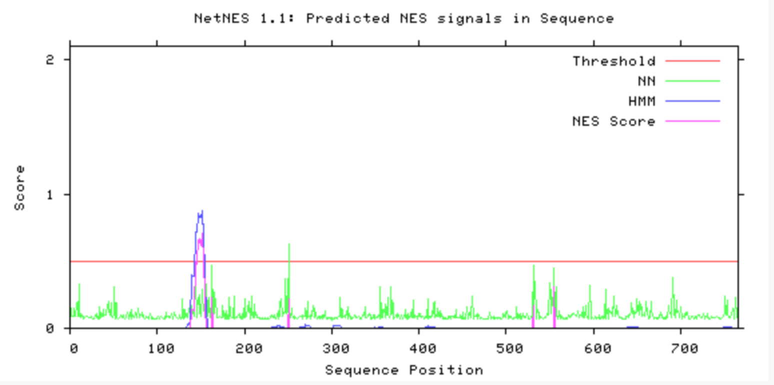

For all analyses, the input consisted of the full-length human SEC23B protein sequence (UniProtKB Q15437, SC23B_HUMAN).

Figure S4: Investigating the dynamics of SEC23B nuclear-cytoplasmic shuttling

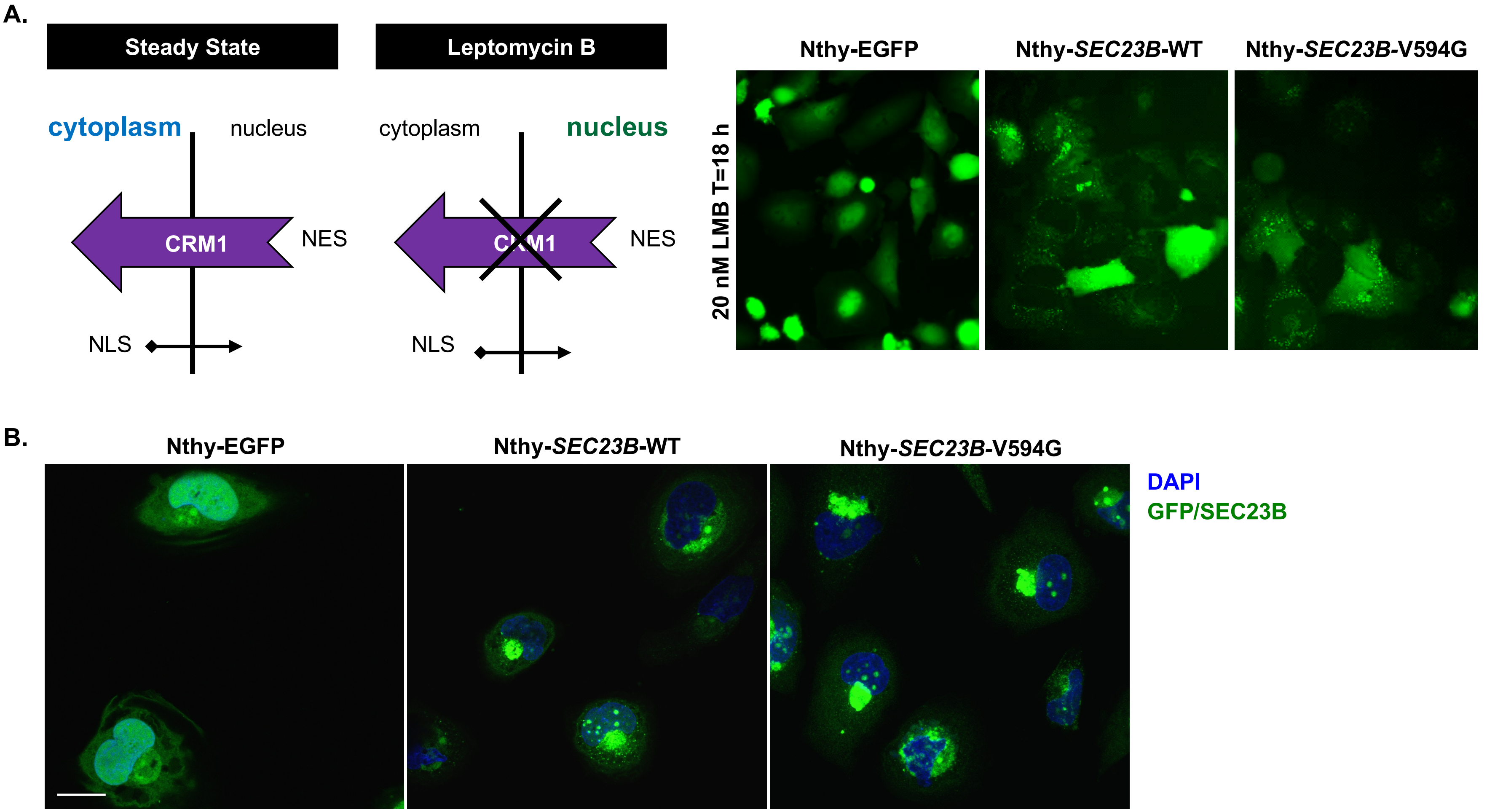

A. Nuclear SEC23B is observed in a subset of Nthy-*SEC23B*-WT and Nthy-*SEC23B*-V594G cells starting at t=2 hours after treatment with Leptomycin B (LMB). Images were taken using a Leica DMI3000B manual inverted microscope (Leica, Buffalo Grove, IL). nM, nanomolar; h, hours.

B. Nucleolar SEC23B is observed in Nthy-*SEC23B*-WT and Nthy-*SEC23B*-V594G cells following treatment for 24 hours with 10 μM of the proteasome inhibitor MG132. Confocal images were taken using a TCS SP8 confocal microscope (Leica). Blue, DAPI; green, SEC23B-EGFP. Scale bars, 25 μm.

Figure S5: SEC23B localizes to nucleolar sub-compartments with MG132 treatment

A.

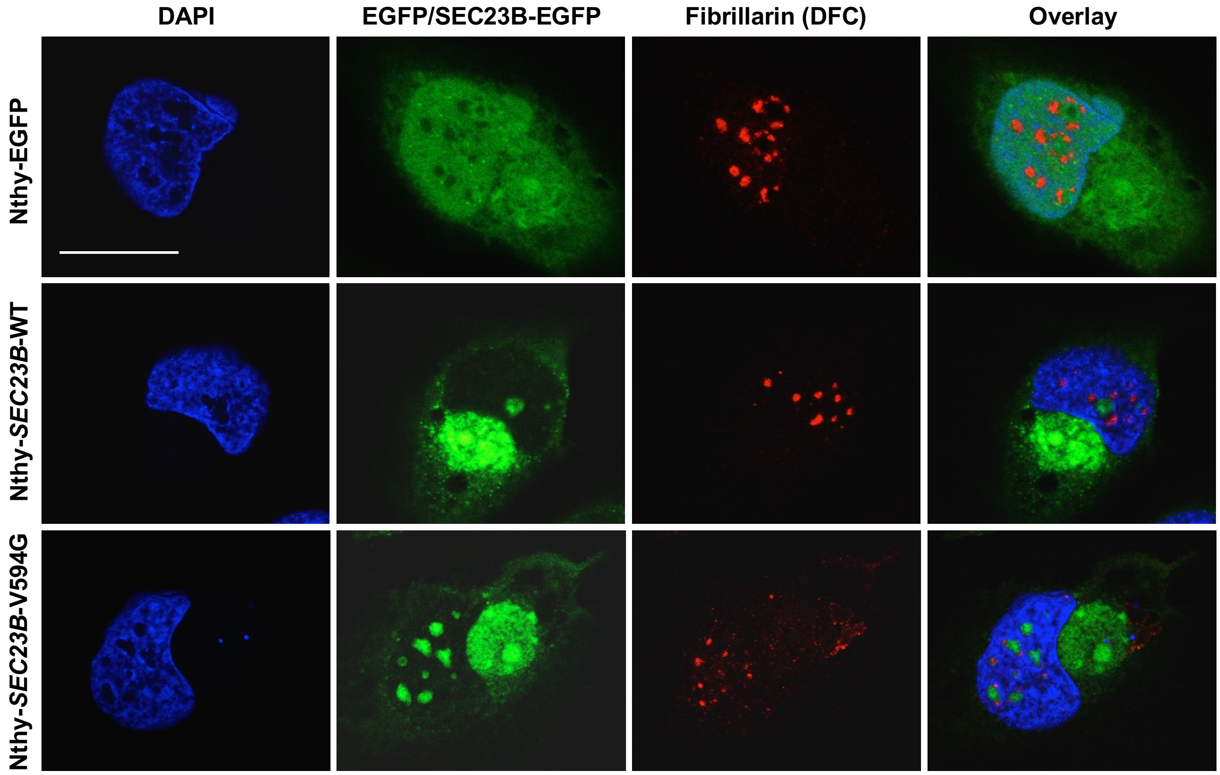

B.

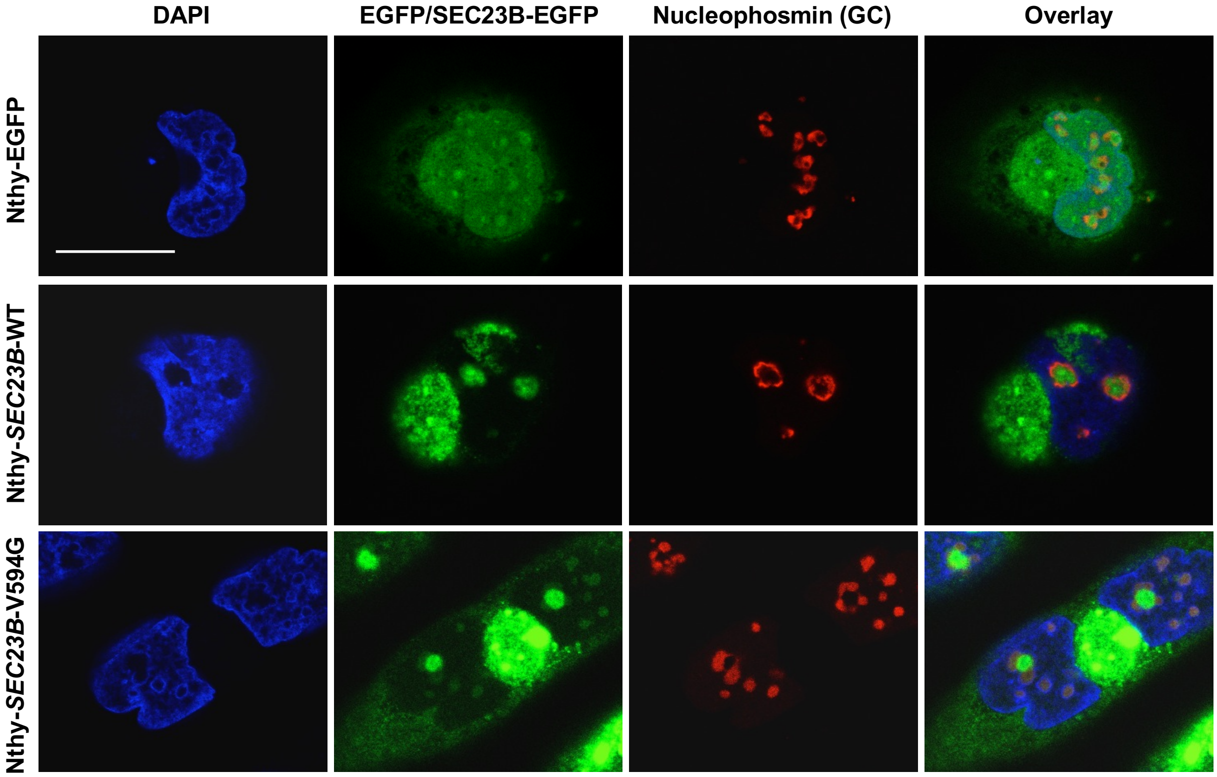

Abbreviations: DFC, dense fibrillar component; GC, granular component. Scale bars, 25 μm.

Figure S6. Proteasome inhibition via Bortezomib causes nucleolar localization of SEC23B

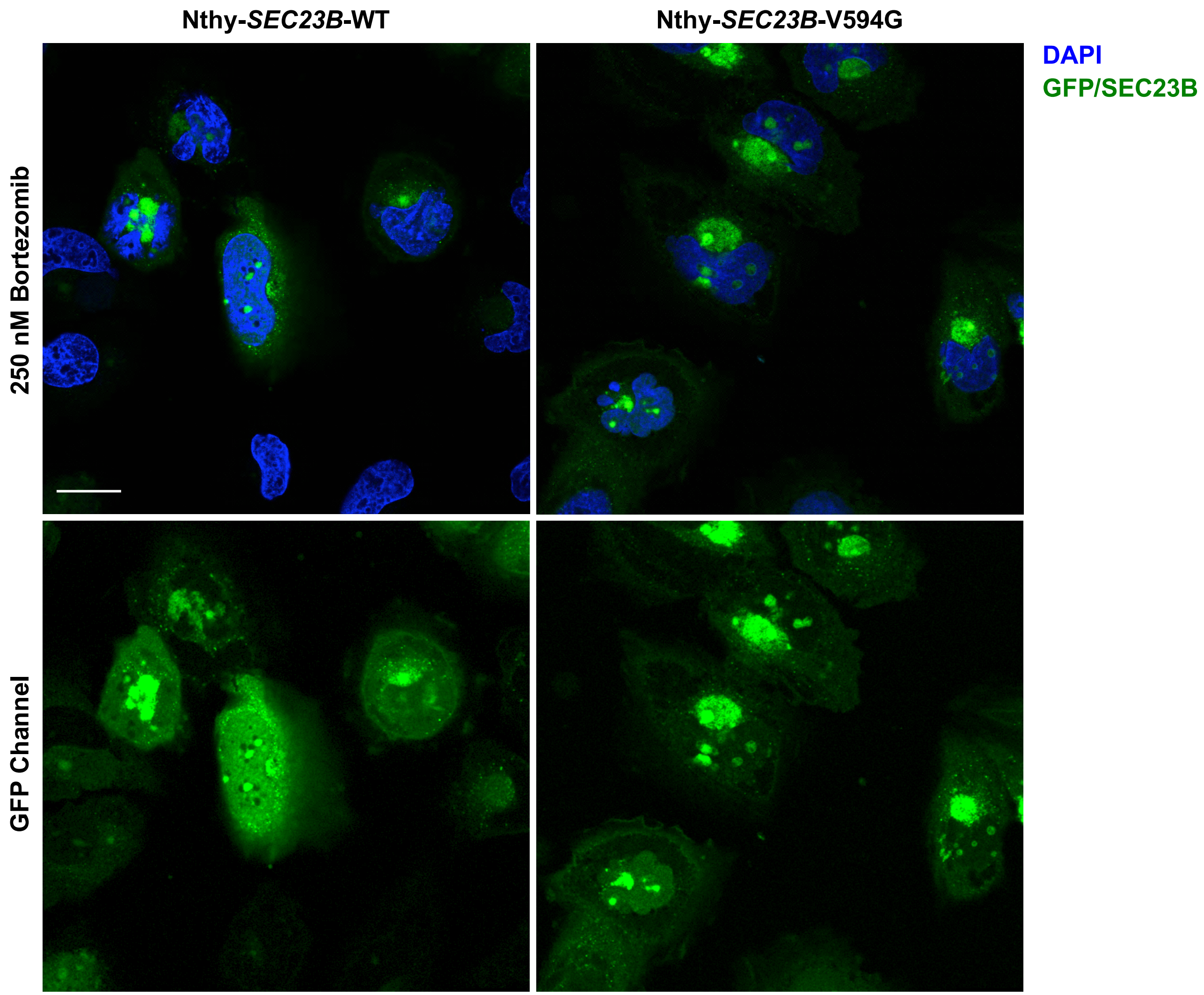

Nucleolar SEC23B is observed in Nthy-*SEC23B*-WT and Nthy-*SEC23B*-V594G cells following treatment for 24 hours with the proteasome inhibitor Bortezomib (BTZ). Confocal images were taken using a TCS SP8 confocal microscope (Leica, Buffalo Grove, IL). Blue, DAPI; green, SEC23B-EGFP. Scale bar, 25 μm.

Figure S7. SEC23B localization to nucleoli is not a generic effect in response to MG132

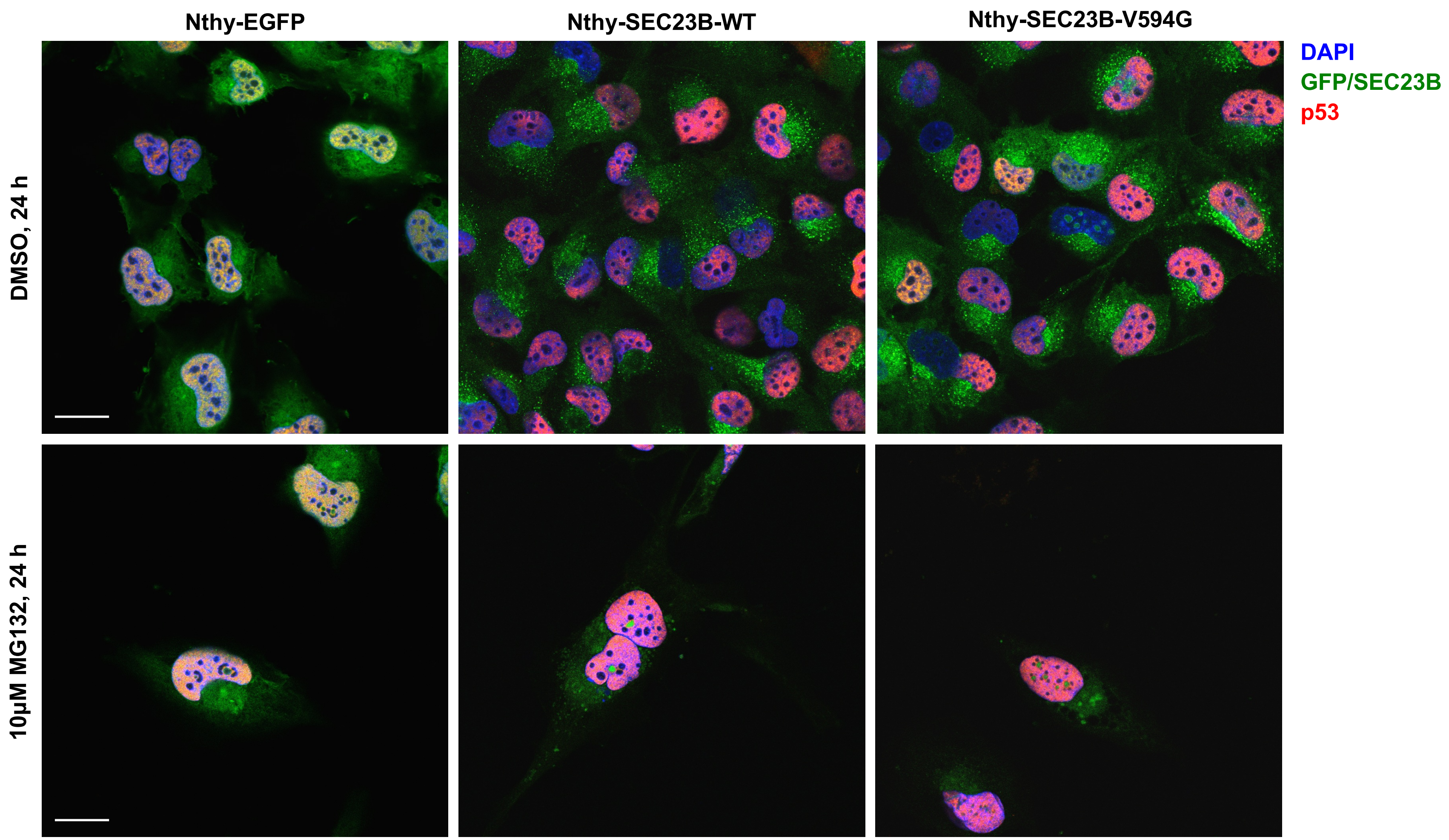

Nthy-EGFP, Nthy-*SEC23B*-WT, and Nthy-*SEC23B*-V594G cells were stained for p53 protein following treatment for 24 hours with the proteasome inhibitor MG132. DMSO mock-treated cells are used as a control. Confocal images were taken using a TCS SP8 confocal microscope (Leica). Blue, DAPI; green, SEC23B-EGFP or EGFP; red, p53. Scale bars, 25 µm.

Figure S8. SEC23B and PTEN overlap in the nucleoli of MG132-treated cells

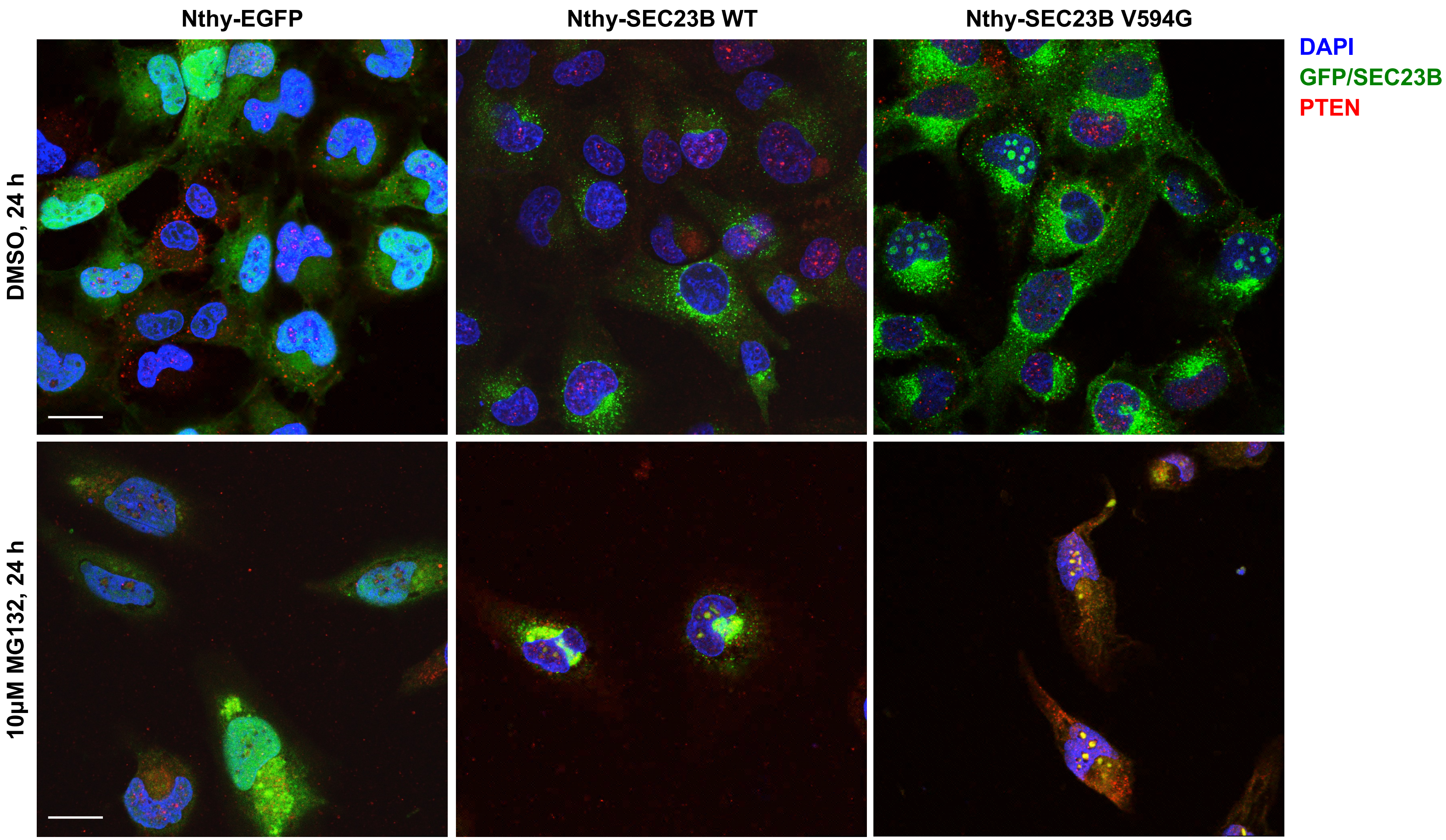

Nthy-EGFP, Nthy-*SEC23B*-WT, and Nthy-*SEC23B*-V594G cells were stained for PTEN protein following treatment for 24 hours with the proteasome inhibitor MG132. DMSO mock-treated cells are used as a control. Confocal images were taken using a TCS SP8 confocal microscope (Leica). Blue, DAPI; green, SEC23B-EGFP or EGFP; red, PTEN. Scale bars, 25 µm.

Figure S9. Aggresome staining at baseline without treatment with MG132

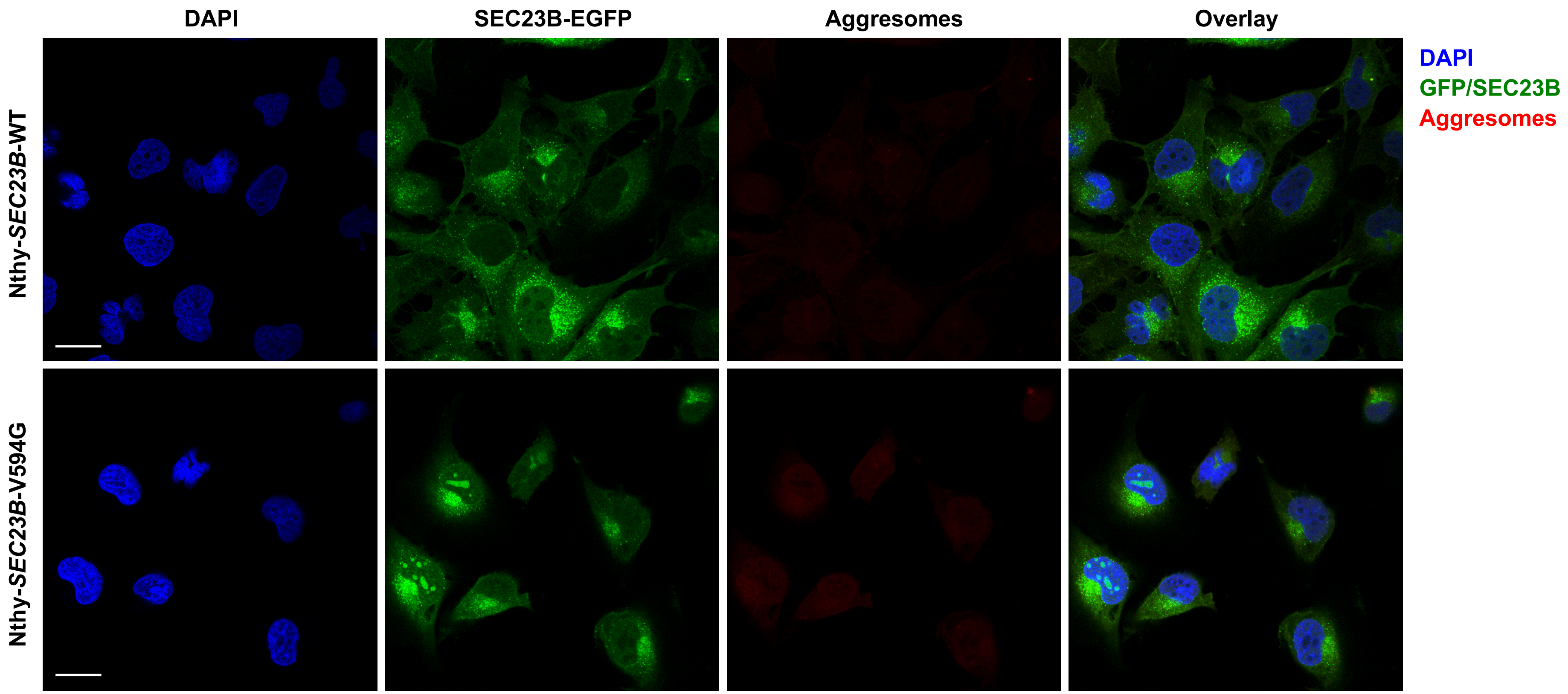

Fluorescent staining of Nthy-*SEC23B*-WT and Nthy-*SEC23B*-V594G thyroid cells for aggresomes (PROTEOSTAT® Aggresome detection kit, Enzo Life Sciences, Farmingdale, NY, USA). Blue, DAPI; green, SEC23B-EGFP or EGFP; red, aggresomes. Scale bars, 25 µm.

Table S1: Primer sequences for the generation of SEC23B NLS and NES truncation constructs

Primer Sequence	Primer Name
ggaagtcgacAATGCTTTGTCAGGTTGATTATC	SEC23B_NLS_72M-SalI-S
ttccGCGGCCGCTTAACAGGCAC	SEC23B_72M/153M-NotI-R
CTGGCTACCAGGACATGCCCtAGTATGAAAACTTCAAGC	SEC23B_NLS_679*-S
GCTTGAAGTTTTCATACTaGGGCATGTCCTGGTAGCCAG	SEC23B_NLS_679*-R
ggaagtcgacAATGTCCCTGAGTCTTCTTC	SEC23B_NES_153M-SalI-S
CATGCCTGGAGGAAGATGACTCCCTGAGTCTTCTTCCTC	SEC23B-deleteNES(144-153)-S
GAGGAAGAAGACTCAGGGAGTCATCTTCCTCCAGGCATG	SEC23B-deleteNES(144-153)-R

Abbreviations: NLS, nuclear localization signal; NES, nuclear export signal.
